# Design and verify a dual modulated metasurface in terahertz range

**DOI:** 10.1038/s41598-020-77051-9

**Published:** 2020-11-16

**Authors:** Min Zhong

**Affiliations:** Guangxi Key Laboratory of Calcium Carbonate Resources Comprehensive Utilization, Hezhou Key Laboratory of Microwave Applied Technology, Hezhou University, Hezhou, 542899 China

**Keywords:** Materials science, Optics and photonics, Physics

## Abstract

A single peak tunable metasurface absorber is proposed and verified in Terahertz (THz) range. This absorption peak is excited by the localized surface plasma (LSP) and dielectric loss modes at resonance frequency 2.98 THz with 83% of amplitude. Three groups of experiments are performed to verify the sensing properties of samples. In the first groups of experiments, temperature is increasing from T = 300 k to T = 400 k, which leads to the absorption peak enhance from 83% (at 2.98 THz) to 93.7% (at 3.5 THz). In the second groups of experiments, samples are covered by ethanol or chloroform (T = 300 k), this absorption peak is also increased and moved to higher frequencies. When temperature and liquid layer are changed simultaneously, samples achieve more intense resonance behaviors in a smaller temperature scale. Finally, this absorption peak is reduced by increasing pump fluence. This proposed tunable metasurface absorber reveals the feasibility of sensing field.

## Introduction

Electromagnetic (EM) metamaterial/metasurface are artificially prepared materials with novel performances^1^. EM metamaterial shows novel electromagnetic properties, for example, negative permittivity, lensing, cloaking, negative refractive index^2–5^. To date, EM metamaterial are popular in various research works and applied in many fields, such as antennas, energy harvesting, lens, and so on^6–8^. Moreover, metasurface devices have attracted the attention of researchers^9–11^. In 2008, the first absorber based on EM metamaterials with metal-dielectric-metal (MDM) structure is designed and verified by Landy et al.^12^. This metamaterial absorber revealed high absorption rate at frequency 11.65 GHz. Since then, perfect metamaterial absorbers become the focus of researchers and are verified in many works. These metamaterial absorbers can achieve unit absorptivity at desired frequencies, and reveal different characteristics, for example, single band^13^, dual band^14^, and multiband^15^, broadband^16^. Nowadays, sensing application based on metamaterial absorbers is an interesting hotspot^17–19^. Since many EM metasurface absorbers can only work at a fixed frequency or modulate by changing structural parameters, their industrial applications are limited. Therefore, it is important to design and prepare a tunable metasurface absorber. To develop a tunable metasurface becomes the preferred solution. At present, researchers have proposed a variety of tunable dielectric layers for developing tunable devices, for example, VO_2_, GST, graphene, liquid crystal, and so on^20–24^.

In this paper, a STO layer is adopted to reveal a tunable metasurface absorber. The permittivity of the STO layer can be controlled through changing temperature. The proposed metasurface absorber adopts a cross metal array and achieves an absorption peak at resonance frequency 2.98 THz. In the first and second groups of experiments, temperature or liquid are changed to reveal the sensitivity of samples. In the third groups of experiments, temperature and liquid are changed simultaneously, more intense resonance behaviors in a smaller dimensional scale are achieved by samples. In the fourth groups of experiments, the absorption peak gradually decreases and moves to low frequencies with the pump fluence increasing. This proposed metasurface absorber exhibits dual tunable properties: thermally controlled and pump fluence controlled.

## Structure and experiments

### Structure

**Figure 1:**
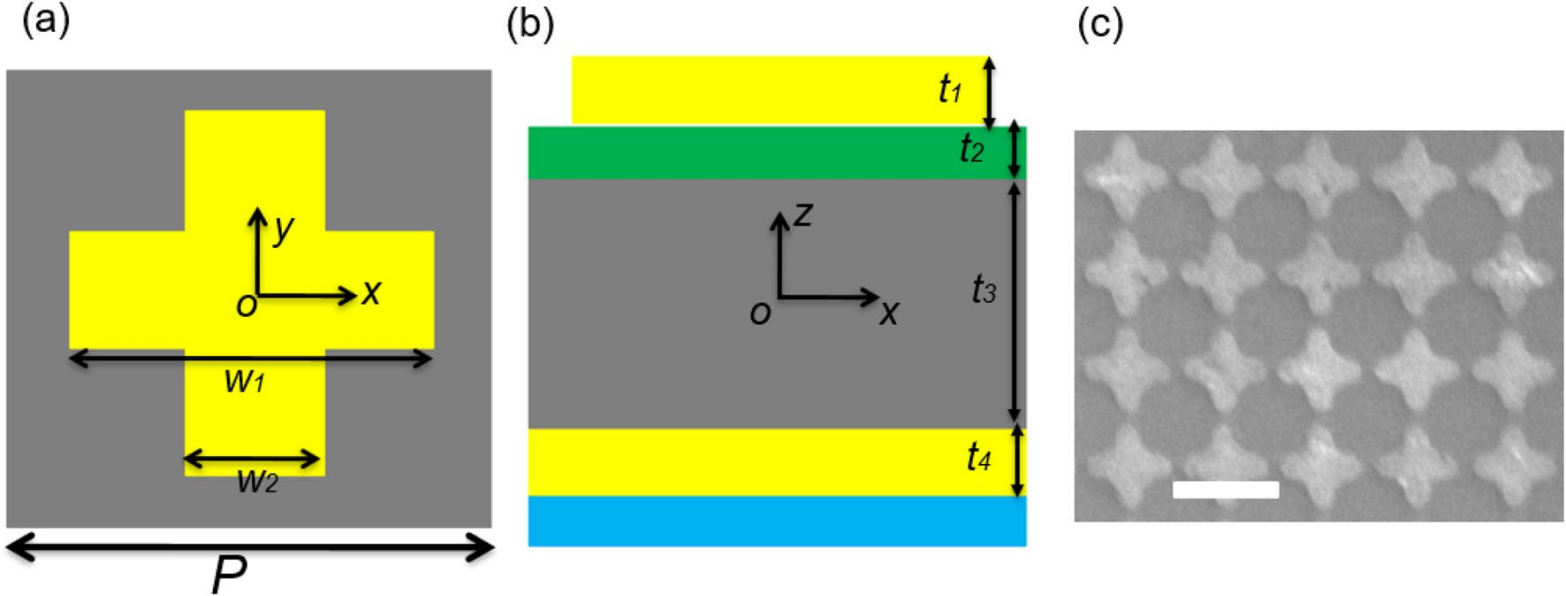
(**a**) Top view of the proposed structure. (**b**) Cross section of the proposed structure. (**c**) SEM of samples. The blue part is air. The yellow parts are metal layers. The gray part is STO layer. The green part is Si layer.

Structure design of the proposed tunable metasurface absorber can be found in Fig. 1a,b. This structure contains of four functional layers. The top layer is a silver layer, a cross array is patterned on the silver layer. This layer is revealed as an electromagnetic resonator. Continuous Si and STO layers are selected as the intermediate dielectric layers. The bottom layer is a continuous silver layer, which plays an electromagnetic eliminator. The geometric parameters of the proposed structure design are shown in Table 1. To reveal the physical mechanism of this metasurface absorber, simulation software HFSS was used. In simulations, both of silver layers are described as following equation:

1 $$ \varepsilon (\omega ) = 1 - \frac{{\omega_{P}^{2} }}{{\omega^{2} - i\omega \gamma_{D} }}. $$

**Table 1:** Geometric parameters.

Parameter	*P*	*w*_1_	*w*_2_	*t*_1_	*t*_2_	*t*_3_	*t*_4_
Value (μm)	10	8.8	2.2	0.1	0.25	2.2	0.25

In Eq. (1), the $\gamma_{D} = 9 \times 10^{13} {\text{s}}^{ - 1}$ is stand to the collision frequency, $\omega_{p} = 1.37 \times 10^{16} {\text{s}}^{ - 1}$ is stand to plasma frequency^25^. Moreover, the STO layer is revealed as the following equation^26,27^:

2 $$ \varepsilon_{w} = \varepsilon_{\infty } + \frac{f}{{w_{o}^{2} - w^{2} - iw\gamma }}. $$

In Eq. (2), the $$\varepsilon_{\infty }$$ is stand to high-frequency bulk permittivity. The $$f = 2.6 \times 10^{6} \,{\text{cm}}^{ - 2}$$ is stand to oscillator strength. The $$ w_{0}$$ is stand to soft mode frequency, reveled in the following Equation:

3 $$ w_{o} (T)\left[ {{\text{cm}}^{ - 1} } \right] = \sqrt {31.2(T - 42.5)} . $$

In Eq. (2), the $\gamma$ is stand to soft mode damping factor:

4 $$ \gamma (T)\left[ {{\text{cm}}^{ - 1} } \right] = - 3.3 + 0.094T. $$

Here, *T* is the simulated temperature.

### Experiments of samples

All of samples can be manufactured according to the following experimental procedures. First, a glass is cleaned and dried, which is set to the substrate of samples. Second, the bottom silver layer is deposited (using ZZL-U400C) on the surface of this glass layer. Third, after the bottom silver layer is cooled, the STO layer can be deposited (using ZZL-U400C) on the bottom silver layer, and the Si layer is deposited on the STO layer. Fourth, the top silver layer is s deposited (using ZZL-U400C) on the surface of the STO layer. Fifth, the cross array is defined (using CABL-9000C) on the top silver layer, as shown in Fig. 1c. Sixth, samples will be cleaned by an ultrasonic (device: VGT-QTD), and the permittivity and loss tangent of liquid layers are measured at room temperature and normal pressure (Solid and liquid dielectric constant tester). Seventh, these achieved samples are bonded to the small heating plate. Through the bottom metal layer, the sample can fully contact the hot plate, which is conducive to temperature change and distribution. Moreover, samples are fixed 10 cm directly below excitation and receiving ports of the Bruker Optics Equinox (55 Fourier spectrometer). Since the transmission is zero, the measured absorption spectrum is achieved as: A = 1 − R. Finally, SEM of samples can be obtained by JSM-7610F.

## Measured results and discussion

### Physical mechanism

**Figure 2:**
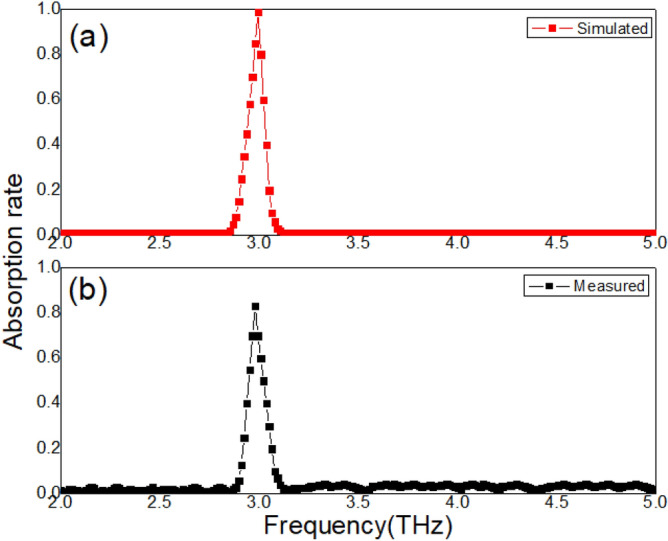
(**a**) Simulated absorption spectrum. (**b**) Measured absorption spectrum.

Measured absorption spectrum of samples at room temperature (*T* = 300 k) and atmospheric pressure is shown in Fig. 2. A single absorption peak can be revealed at resonance frequency 2.98 THz. The maximum absorption rate reaches 83%. In this paper, the full width at half maximum of band is defined as follows:

5 $$ \Delta f = f_{H} - f_{L} . $$

Here, $f_{H}$ is the frequency at which the absorption rate decreases from the maximum to 50%, and $f_{L}$ is the frequency at which the absorption rate increases from the minimum to 50%. In Fig. 2, the measured $\Delta f$ is 0.06 THz. Corresponding simulated absorption spectrum is also can be found in Fig. 2. In simulations, boundary conditions are set based on the reported work^28^. The simulation results are in good agreement with the experimental results, which proves that simulation conditions are basically effective. To evaluate the performance of the absorber, quality factor Q is applied in this paper:

6 $$ Q = \frac{{f_{o} }}{\Delta f}. $$

**Figure 3:**
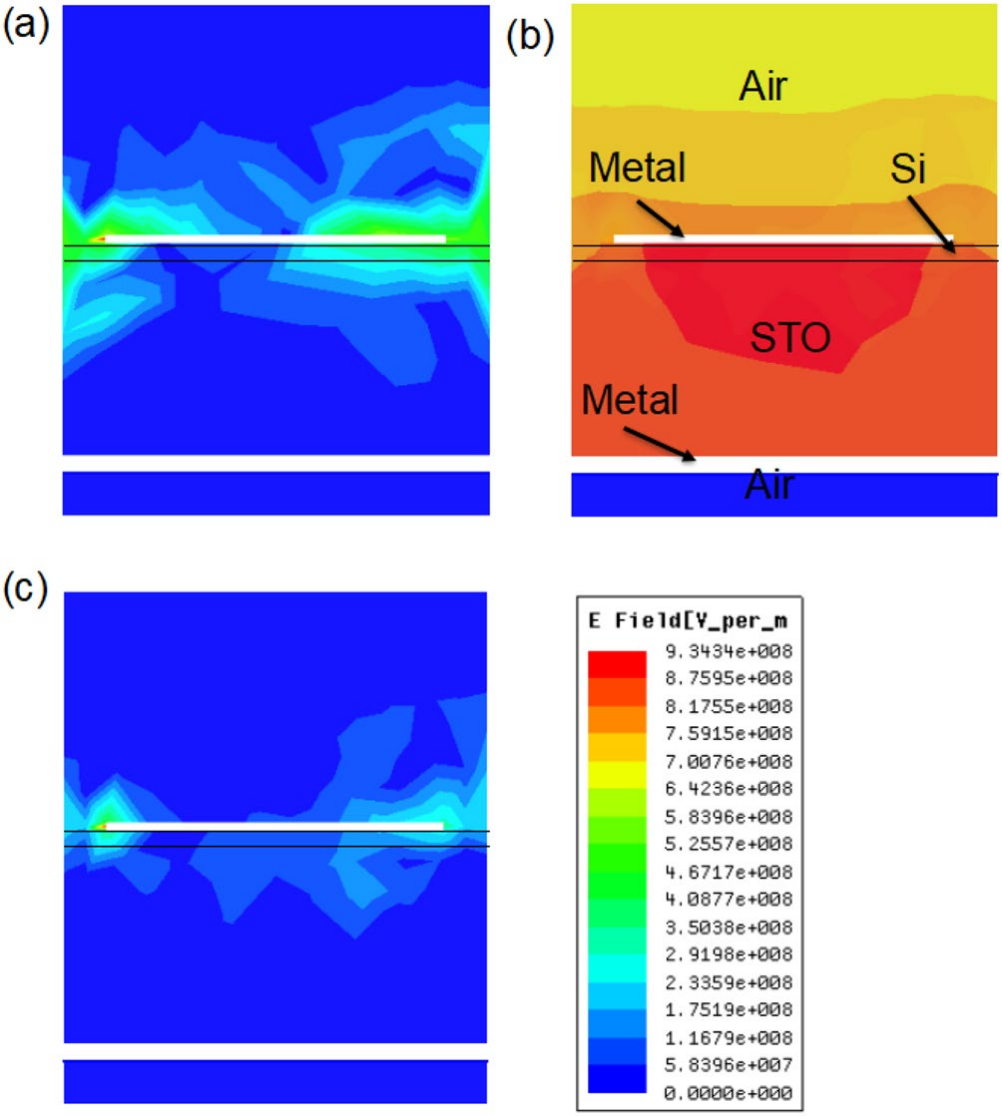
(**a**) Simulated electric fields at the frequency 2.7 THz. (**b**) Simulated electric fields at the resonance frequency 2.98 THz. (**c**) Simulated electric fields at the frequency 3.2 THz.

Based on these measured results, the measured Q is achieved as 49.7. This high quality factor Q indicates that the proposed samples have potential value for sensing applications. To understand the physical mechanism in Fig. 2, electric field is calculated at resonance frequency 2.98 THz, as shown in Fig. 3. At 2.98 THz, the simulated electric fields are mainly distributed on the cross array and inside the Si and STO layers, as shown in Fig. 3b. LSP modes are excited on the metal cross array. This absorption loss of electromagnetic waves is mainly caused by two factors at 2.98 THz: the LSP modes on the metal cross array and the dielectric loss mode inside the Si and STO layers, which result in the peak in Fig. 2. When simulated frequencies are deviated from the resonance frequency, both modes are failed, as shown in Fig. 3a,c.

### Effect of temperature

**Figure 4:**
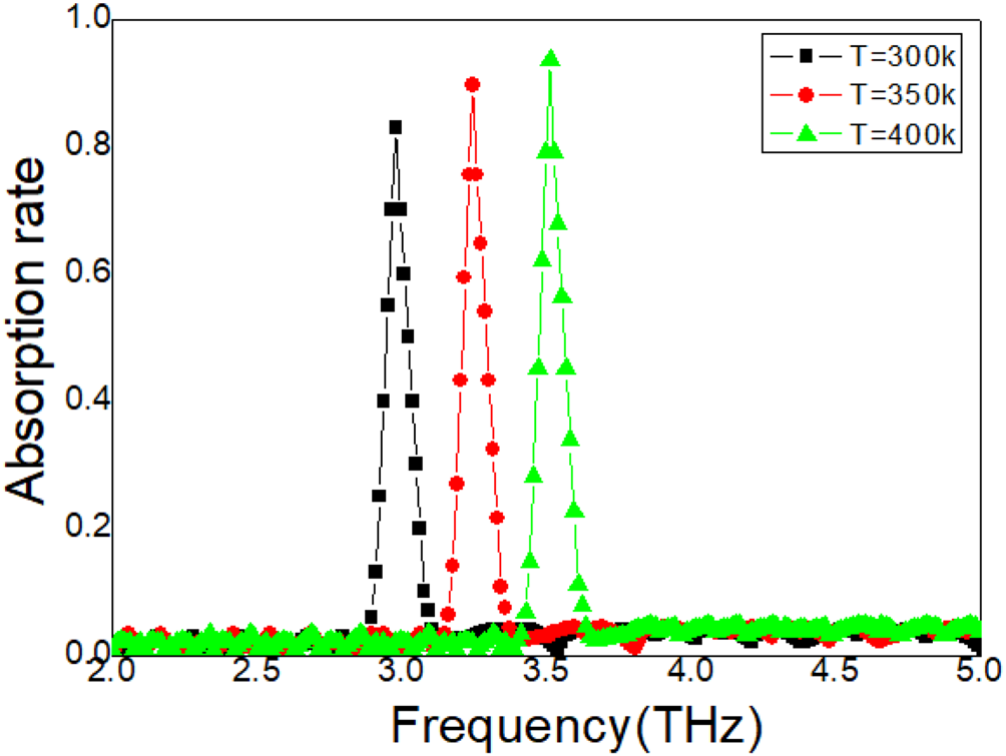
Measured absorption spectrum with different temperature.

**Figure 5:**
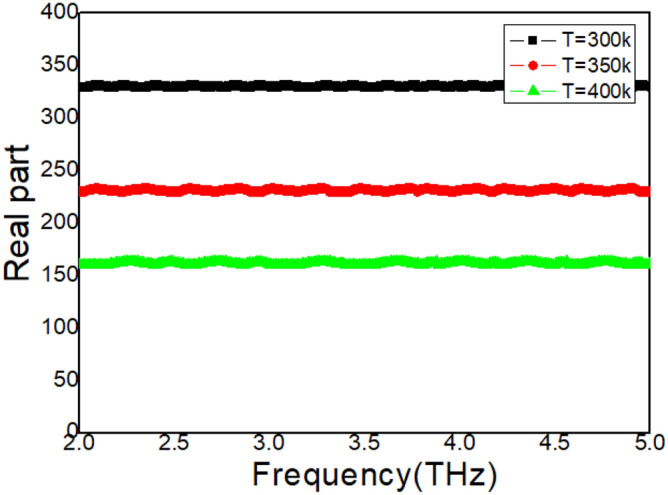
Measured real part of the permittivity with different temperature.

In Fig. 2, an absorption peak is revealed with high quality factor Q by the samples. Therefore, it is necessary to reveal the sensing feasibility of these samples. It is found that the absorption peak is excited by the LSP modes on the cross array and the dielectric loss mode inside the STO layer, as shown in Fig. 3. The resonance properties (resonance frequency and amplitude) are temperature sensitive. This is due to the permittivity of this STO layer is directly related to temperature according to the Eq. (2). In the first experiments, temperature is enhanced from 300 to 400 k, other experimental conditions remain unchanged. The measured results are achieved in Fig. 4. This peak is shifted from 2.98 THz to 3.51 THz with temperature increasing. Moreover, the maximum absorption rate is also enhanced. The measured quality factors Q are: 49.7, 54.1, and 58.5 with different temperature. These measured results reveal that the samples have potential value for thermal sensing. To understand this temperature-dependence property in Fig. 4, the permittivity of this STO layer is measured, as shown in Fig. 5. Using dielectric constant measuring equipment ZJD-C, the resonance point frequency is set to automatic search, the Q value range is set to automatic shifting, measurement sensitivity: < 30 mV, preheating for more than 30 min, the fixture is in full contact with the sample, and two wiring posts are connected, thereby measuring the permittivity of the STO layer. The permittivity of liquid is measured at room temperature and normal pressure. It is obvious that the real part of permittivity is decreased with temperature increasing. According to the perturbation theory, the permittivity of STO layer and the resonance frequency is revealed as follows^29–31^:

7 $$ \frac{\Delta \omega }{\omega } = \frac{{\omega - \omega_{o} }}{{\omega_{o} }} \approx \frac{{ - \iiint_{V} {[(\Delta \vec{\varepsilon } \bullet \vec{E}) \bullet \vec{E}_{o}^{ * } + (\Delta \vec{\mu } \bullet \vec{H}) \bullet \vec{H}_{o}^{*} ]dV}}}{{\iiint {(\varepsilon \left| {\vec{E}_{o} } \right|^{2} + \mu \left| {\vec{H}_{o} } \right|^{2} )dV}}}, $$

where, $\Delta \vec{\varepsilon }$ is set to difference of permittivity, $\Delta \vec{\mu }$ is set to difference of permeability. As the measured temperature increase, the real part of permittivity can be continuously reduced. Therefore, the difference of permittivity $\Delta \vec{\varepsilon }$ is a negative value. This negative difference of permittivity leads to the resonance frequency increase and the absorption peak shift to higher frequencies based on Eq. (7).

The sensitivity of this metasurface to temperature is expressed by the following formula:

8 $$ S = \left| {\frac{\Delta f}{{\Delta T}}} \right|. $$

Here, $\Delta f$ is the frequency difference, and $\Delta T$ is temperature difference. Two high sensitivities can be obtained by metasurface samples: $S_{{T = 350{\text{K}}}} = 3.9 \times 10^{9} \,{\text{Hz}}/{\text{K}}$, and $S_{{T = 400{\text{K}}}} = 3.6 \times 10^{9} \,{\text{Hz}}/{\text{K}}$. At the same time, figure of merit (FOM) is also applied in revealing the performance of this metasurface samples:

9 $$ FOM = Max\left| {\frac{dI(f)/dT(f)}{{I(f_{o} )}}} \right|, $$

where, $I(f_{o} )$ stands for the intensity where FOM reaches the maximum, $dI(f)/dT(f)$ stands for the intensity difference with the $dT(f)$. Two FOM are achieved: $FOM_{{T = 350{\text{K}}}} = 3426$, and $FOM_{{T = 400{\text{K}}}} = 3207$.

### Influence of the refractive index

**Figure 6:**
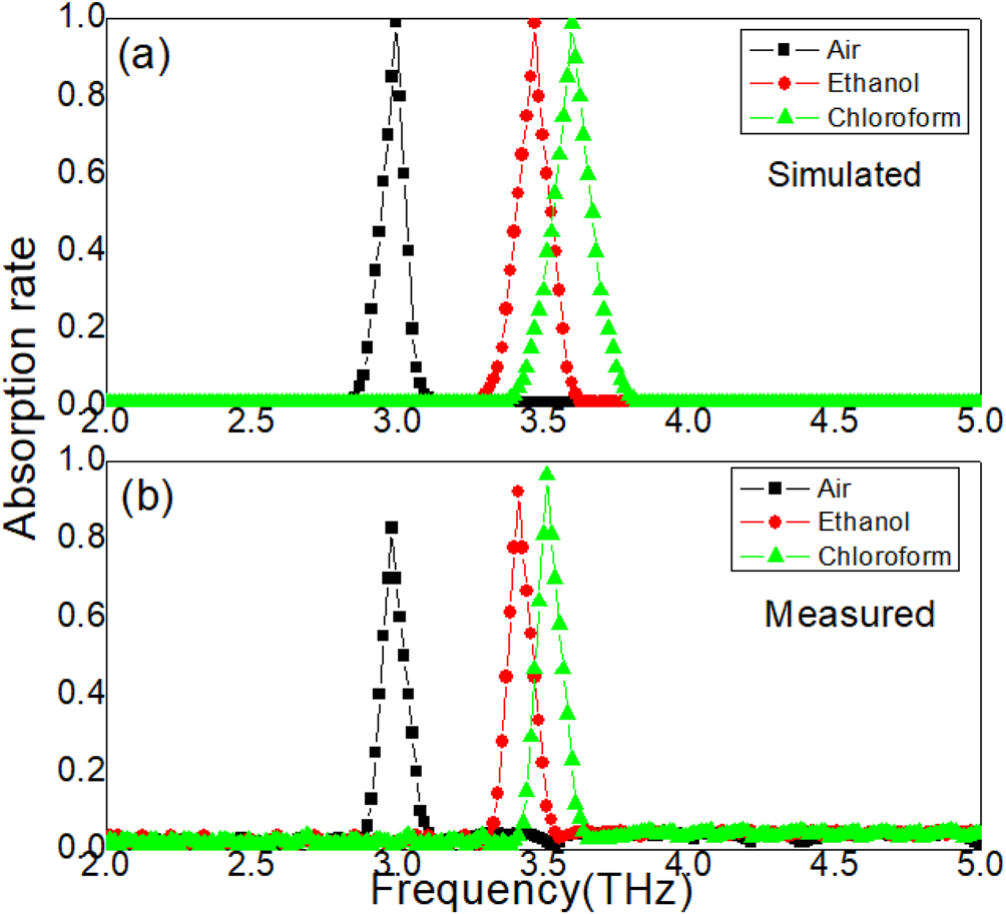
(**a**) The simulated absorption spectrum with different liquid at room temperature. (**b**) The measured absorption spectrum with different liquid at room temperature.

The absorption peak in Fig. 2 is related to the LSP mode resonance, which provides the refractive index sensing feasibility of samples. This is because the resonance properties of the LSP modes are sensitive to changes in environmental media. Therefore, in the second group of experiments, two kinds (ethanol and chloroform) of liquid (temperature is T = 300 k) are covered on the surface of the samples, respectively. Samples are placed in a Petri dish. The electromagnetic wave probe is located 3 cm directly above the sample, and the thickness of the liquid layer is 0.5 mm. The measured absorption spectrum is achieved in Fig. 6. When the samples are covered by ethanol, the absorption peak is enhanced to 92.3%. Moreover, the resonance frequency is found at 3.41 THz. When ethanol is cleaned, the samples are cover by chloroform. The absorption peak is increased from 83 to 96.6%, and the resonance frequency is shifted from 2.98 to 3.51 THz. The refractive index of environmental media is changed with different liquid, which is directly related to the resonance intensity of the LSP mode.

### Influence of both temperature and refractive index

**Figure 7:**
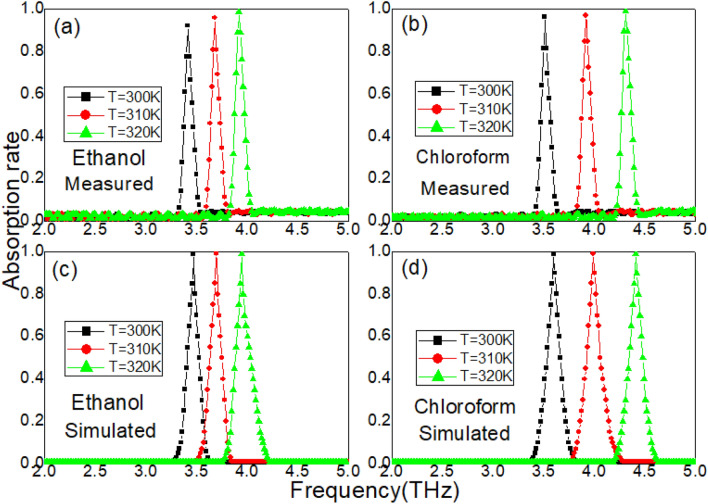
(**a**) The measured absorption spectrum with different temperature (ethanol layer). (**b**) The measured absorption spectrum with different temperature (chloroform layer). (**c**) The simulated absorption spectrum with different temperature (ethanol layer). (**d**) The simulated absorption spectrum with different temperature (chloroform layer).

In the first and second groups of experiments, the temperature or refractive index are changed separately. Since the absorption peak in Fig. 2 is directly related to the LSP mode and the STO layer. It is necessary to verify the resonance behavior of the absorption peak when the temperature and refractive index are changed simultaneously. In the third group of experiments, the samples are covered by ethanol or chloroform, respectively. At the same time, temperature is set to be 300 k, 310 k, and 320 k. The metasurface samples are placed on a hot plate. The electromagnetic wave probe is located 3 cm directly above the sample, and the thickness of liquid is 0.5 mm. The measured absorption spectrum is achieved in Fig. 7. It can be seen that the absorption peak exhibits stronger resonance behaviors than that in Figs. 4 and 6. When the samples are covered by ethanol, the maximum absorption rates are 92.3%, 96%, and 99.1%. The resonance frequencies are 3.41 THz, 3.68 THz, and 3.92 THz. When ethanol is replaced by chloroform, the maximum absorption is enhanced from 96.6 to 99.2% for 20 k temperature changes. Moreover, the absorption peak is shifted from 3.51 to 4.31 THz with temperature increasing. These measured results in Fig. 7 are related to the refractive index-temperature resonance property of ethanol or chloroform. The refractive indices of both liquid can be achieved as follows:

10 $$ n = n_{0} - C_{T} (T - T_{0} ). $$

In the above Equation, $T_{0} { = }300{\text{k}}$ is set to room temperature. $C_{T}$ is set to refractive index temperature coefficient. $n_{0}$ is set to refractive index of ethanol or chloroform. When the room temperature is 300 k, $n_{0}$ for ethanol is close to 1.36048, and for chloroform is close to 1.44 ^32^. When temperature is changed, two direct phenomena are obtained: the permittivity of the STO layer is reduced according to Eq. (2) and the refractive index of both liquid is also changed according to Eq. (8). A small measured temperature change results in more severe resonance behaviors than that in Figs. 4 and 6. Based on formulas (8–9), two high sensitivities are achieved by metasurface samples for the chloroform case: $S_{{T = 310{\text{K}}}}^{Chloroform} = 3.78 \times 10^{10} \,{\text{Hz}}/{\text{K}}$, and $S_{{T = 320{\text{K}}}}^{Chloroform} = 3.91 \times 10^{10} \,{\text{Hz}}/{\text{K}}$. For the ethanol case, two high sensitivities are achieved: $S_{{T = 310{\text{K}}}}^{Ethanol} = 1.94 \times 10^{10} \,{\text{Hz}}/{\text{K}}$, and $S_{{T = 310{\text{K}}}}^{Ethanol} = 1.87 \times 10^{10} \,{\text{Hz}}/{\text{K}}$. The proposed metasurface samples are shows higher sensitivities when the temperature is increased, as shown in Fig. 7.

### Influence of the Si layer

**Figure 8:**
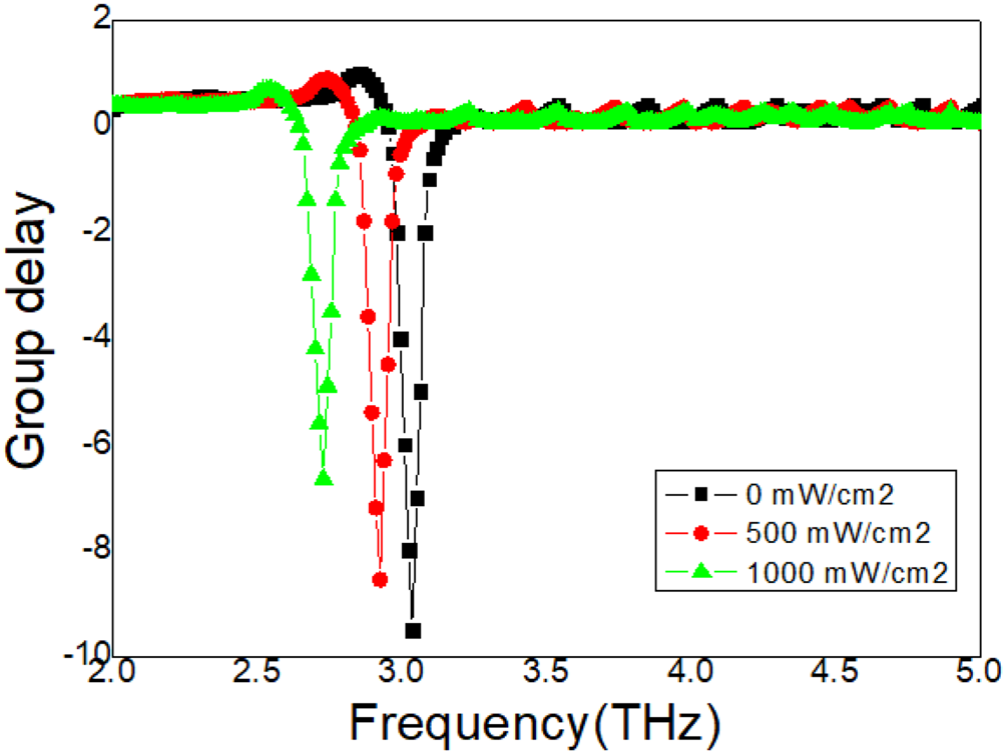
The measured group delay spectrum with different pump fluence.

**Figure 9:**
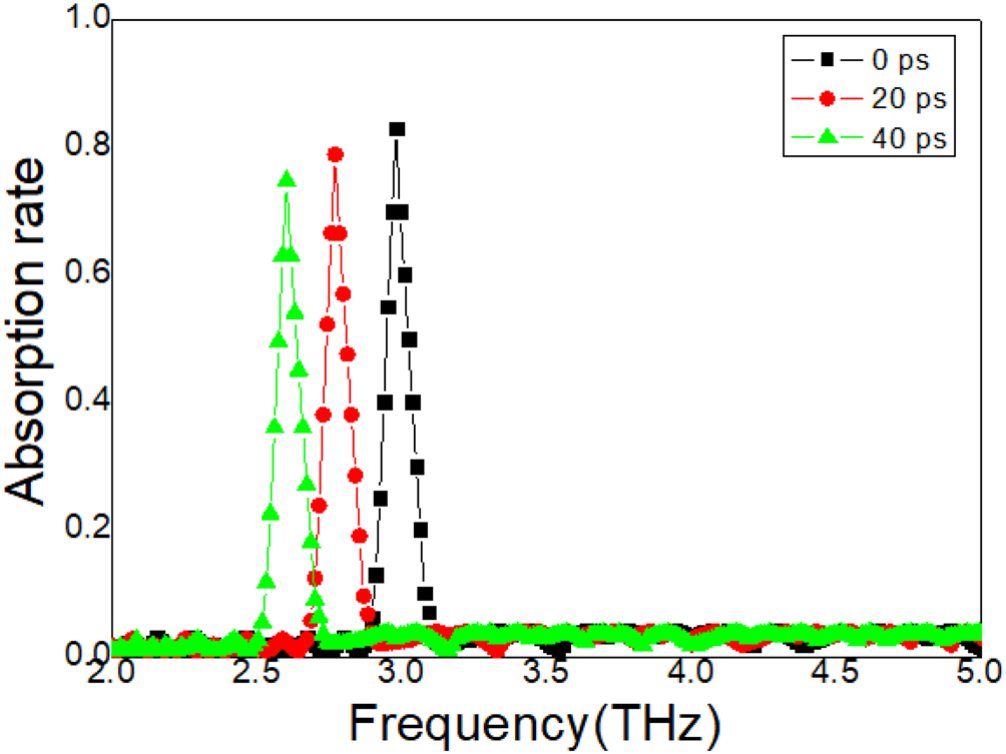
The measured absorption spectrum with different time delayer.

The influence of the sensitivity of the Si layer on the properties of the metasurface samples also needs to be revealed. At the beginning, the pump fluence is 0 mW/cm^2^, a group delay dip appears at the resonance frequency 3.41 THz, seen the black curve in Fig. 8. The group delay reaches − 0.9. As the pump fluence increases, the group delay gradually decreases and moves toward lower frequencies, as shown in Fig. 8. Moreover, the influence of the time delay on the Si layer is also measured, as shown in Fig. 9. For the original state, the time delay is 0 ps, the measured waves reach the metasurface samples are earlier than the laser pump, an absorption peak is achieved at 3.41 THz, seen the black curve in Fig. 9. As the time delay increases, the absorption peak gradually decreases and moves to low frequencies. This resonance behavior is similar to that in Fig. 8. Actively tunable metasurfaces are the focus of researchers^20–24^. A variety of modulation metasurface resonance properties are recommended and confirmed, such as electrical excitation, optical pumping, and lasers^33–35^. These modulation methods have greatly expanded the application range of tunable metasurfaces. These proven tunable metasurfaces can only be actively controlled through a single method^20–24,33–35^. The measurement results shown in Figs. 4 and 9 show that this proposed metasurface can be actively modulated using two strategies, temperature and pump fluence, which is the main advantage of this structure.

## Conclusion

A tunable metasurface absorber in THz range is verified. An absorption peak is found at resonance frequency 2.98 THz with 83% of amplitude. This absorption peak is sensitive to temperature and refractive index changes of the environmental medium because it is excited by the LSP and dielectric loss modes. In the first and second groups of experiments, temperature or liquid are changed, respectively. This absorption peak is enhanced and moved towards higher frequencies. When the temperature and liquid are changed simultaneously, samples achieve more intense resonance behaviors in a smaller dimensional scale. Moreover, the absorption peak can be also controlled by pump fluence. This proposed tunable metasurface absorber shows dual tunable properties: thermally controlled and pump fluence controlled.
